# The Quantile Frailty Index: A Cutpoint-Free Approach to Biomarker-Based Health Assessment

**DOI:** 10.1093/geroni/igaa057.582

**Published:** 2020-12-16

**Authors:** Garrett Stubbings, Spencer Farrell, Arnold Mitnitski, Kenneth Rockwood, Andrew Rutenberg

**Affiliations:** Dalhousie University, Halifax, Nova Scotia, Canada

## Abstract

We develop a frailty index (FI) from continuous valued biomarker measurements that does not use thresholds to binarize deficits. In this work we construct a quantile frailty index (FI-Q) directly from risk quantiles, without binarizing the deficits. FI-Q is the average risk quantile for an individual in the population with respect to the set of measured biomarkers. We show that FI-Q predicts adverse health outcomes better than either a quantile-based cutpoint approach or an FI-Lab method used in previous studies. We also address practical questions such as how to use longitudinal data. We use data from the English Longitudinal Study of Ageing (ELSA) for longitudinal analysis and data from the National Health and Nutrition Examination Survey (NHANES) and the Canadian Study of Health and Aging (CSHA) to compare predictive value of FI-QM with previous FI-Lab studies.

